# Albumin administration is associated with acute kidney injury in cardiac surgery: a propensity score analysis

**DOI:** 10.1186/s13054-014-0602-1

**Published:** 2014-11-14

**Authors:** Anne Julie Frenette, Josée Bouchard, Pascaline Bernier, Annie Charbonneau, Long Thanh Nguyen, Jean-Philippe Rioux, Stéphan Troyanov, David R Williamson

**Affiliations:** Department of Pharmacy, Hôpital du Sacré-Coeur de Montreal, 5400 Blvd Gouin West, H4J 1C5 Montreal, QC Canada; Faculty of Pharmacy, University of Montreal, 2900 Blvd Edouard-Monpetit, H3T 1J4 Montreal, QC Canada; Research Center, Hôpital du Sacré-Coeur de Montréal, 5400 Blvd Gouin West, H4J 1C5 Montreal, QC Canada; Department of Nephrology, Hôpital du Sacré-Coeur de Montreal, 5400 Blvd Gouin West, H4J 1C5 Montreal, QC Canada; Faculty of Medicine, University of Montreal, 2900 Blvd Edouard-Monpetit, H3T 1J4 Montreal, QC Canada

## Abstract

**Introduction:**

The risk of acute kidney injury (AKI) with the use of albumin-containing fluids compared to starches in the surgical intensive care setting remains uncertain. We evaluated the adjusted risk of AKI associated with colloids following cardiac surgery.

**Methods:**

We performed a retrospective cohort study of patients undergoing on-pump cardiac surgery in a tertiary care center from 2008 to 2010. We assessed crystalloid and colloid administration until 36 hours after surgery. AKI was defined by the RIFLE (risk, injury, failure, loss and end-stage kidney disease) risk and Acute Kidney Injury Network (AKIN) stage 1 serum creatinine criterion within 96 hours after surgery.

**Results:**

Our cohort included 984 patients with a baseline glomerular filtration rate of 72 ± 19 ml/min/1.73 m^2^. Twenty-three percent had a reduced left ventricular ejection fraction (LVEF), thirty-one percent were diabetics and twenty-three percent underwent heart valve surgery. The incidence of AKI was 5.3% based on RIFLE risk and 12.0% based on the AKIN criterion. AKI was associated with a reduced LVEF, diuretic use, anemia, heart valve surgery, duration of extracorporeal circulation, hemodynamic instability and the use of albumin, pentastarch 10% and transfusions. There was an important dose-dependent AKI risk associated with the administration of albumin, which also paralleled a higher prevalence of concomitant risk factors for AKI. To address any indication bias, we derived a propensity score predicting the likelihood to receive albumin and matched 141 cases to 141 controls with a similar risk profile. In this analysis, albumin was associated with an increased AKI risk (RIFLE risk: 12% versus 5%, *P* = 0.03; AKIN stage 1: 28% versus 13%, *P* = 0.002). We repeated this methodology in patients without postoperative hemodynamic instability and still identified an association between the use of albumin and AKI.

**Conclusions:**

Albumin administration was associated with a dose-dependent risk of AKI and remained significant using a propensity score methodology. Future studies should address the safety of albumin-containing fluids on kidney function in patients undergoing cardiac surgery.

## Introduction

Acute kidney injury (AKI) following cardiac surgery is prevalent and associated with considerable morbidity and mortality [1,2]. Perioperative resuscitation solutions in intensive care units shifted from crystalloids toward colloids in the hope of facilitating intravascular volume repletion [3,4]. However, synthetic starches are now recognized as an independent risk factor for AKI in critically ill patients [5,6]. The Kidney Disease Improving Global Outcome (KDIGO) Clinical Practice Guidelines for AKI favor isotonic crystalloids over colloids in patients at risk for or presenting with AKI in the absence of hemorrhagic shock [7]. Whether this applies equally to different synthetic starches and albumin is uncertain.

Study results regarding administration of albumin-containing fluids have been conflicting. Hyperoncotic albumin has demonstrated a clear benefit in patients with cirrhosis [8,9]. However, in patients with shock, hyperoncotic albumin has been associated with a fivefold increased risk of AKI [10]. A recent European consensus statement recommends withholding hyperoncotic albumin use in critically ill patients outside of clinical trials [10,11]. The recently published Albumin Italian Outcome Sepsis (ALBIOS) Study, a randomized controlled trial (RCT) on the effect of hyperoncotic albumin (20%) versus crystalloids in hypoalbuminemic patients with severe sepsis and septic shock, did not show any difference in mortality and AKI between the groups [12]. However, the timing of AKI in relation to albumin administration >was not detailed. The use of iso-oncotic albumin in a heterogeneous population of critically ill patients has also been studied in a large, multicenter RCT: the Saline versus Albumin Fluid Evaluation (SAFE) study. In that RCT, the investigators concluded that iso-oncotic albumin administration is safe from a survival perspective [4,13] and found no differences in organ dysfunction or duration of renal replacement therapy (RRT). However, kidney function was not independently reported, and only severe cases of AKI were collected. No data specifically on the safety of albumin as it relates to kidney function after cardiac surgery exist.

In 2009, we reported a dose-dependent risk of AKI using pentastarch 10% (250 kDa/0.45) following cardiac surgery [6]. Since then, our practice has shifted toward the use of hydroxyethyl starch (HES) 6% (130 kDa/0.4) and albumin for volume expansion in unstable patients, and more recently it has shifted toward the use of crystalloids with or without albumin. In the present study, we hypothesized that both synthetic starches and albumin-containing solutions are independently associated with AKI in a dose-dependent fashion following cardiac surgery.

## Materials and methods

### Study design and setting

We conducted a retrospective cohort study of patients who underwent on-pump cardiac surgery between 1 January 2008 and 31 December 2010 at the Hôpital du Sacré-Coeur de Montréal, a tertiary care teaching hospital. The intensive care unit is a 24-bed mixed medical-surgical and trauma unit. The research ethics board of the Hôpital du Sacré-Coeur de Montréal approved the study protocol and waived the need for consent from the study participants.

### Patients

We included all adult patients who required either coronary artery bypass graft (CABG) and/or valve replacement and who were hospitalized at least 96 hours after surgery. Patients known to have end-stage renal disease (ESRD) or AKI (defined on the basis of the RIFLE (risk, injury, failure, loss and end-stage kidney disease) serum creatinine criterion), kidney transplant, autoimmune kidney disease or kidney cancer were excluded as being those who had undergone urgent aortic surgeries. ESRD was defined as a baseline creatinine clearance inferior to 15 ml/min/1.73 m^2^ or treatment with dialysis [14]. Data were obtained from patients’ charts on a prevalidated case report form by three investigators (PB, AC and LTN).

### Data collection

We recorded baseline risk factors for AKI, including patient demographics, history of hypertension, diabetes, peripheral vascular disease, stroke, recent myocardial infarction (within 7 days before surgery), left ventricular ejection fraction (LVEF) and preoperative hemoglobin level. We assessed any use of angiotensin-converting enzyme inhibitors and angiotensin receptor blockers, nonsteroidal anti-inflammatory drugs, contrast agents and diuretics within 5 days before surgery. Preoperative kidney function was estimated using the Chronic Kidney Disease Epidemiology Collaboration (CKD-EPI) creatinine equation [15].

We collected type of surgery (elective or urgent; CABG only or heart valve surgery), as well as perioperative variables, including the Sequential Organ Failure Assessment (SOFA) score for cardiovascular system immediately after surgery [16], use of an intra-arterial balloon pump (IABP), duration of extracorporeal circulation (ECC), and the need for reintervention. We recorded the amount of crystalloids, colloids and blood product transfusions, including red blood cells (RBCs), platelets and fresh frozen plasma (FFP) administered from surgery until 36 hours postoperatively. Colloids consisted of pentastarch 10% (250 kDa/0.45 Pentaspan; Bristol-Myers Squibb, Montreal, QC, Canada), 6% HES (130 kDa/0.4 Voluven™; Fresenius Kabi, Richmond Hill, ON, Canada) and human albumin 5% and 25% (Alburex™ 5 and 25; CSL Behring, Ottawa, ON, Canada). Synthetic colloids are expressed as the patient’s weight in milliliters per kilogram. The amount of albumin 5% and 25% given was converted in absolute grams of albumin, added, and expressed by the patient’s weight in grams per kilogram.

### Definitions

Glomerular filtration rate (GFR) was estimated by using the CKD-EPI formula [15]. Postoperative AKI was assessed using the RIFLE serum creatinine criterion as described in our previous publication [6]. We also assessed the Acute Kidney Injury Network (AKIN) stage 1 serum creatinine criterion [17]. The highest postoperative serum creatinine within 96 hours after surgery was compared with the preoperative creatinine value. Missing values were found in less than 1% of individuals for each variable.

### Statistical analysis

Normally distributed variables were expressed as mean ± standard deviation and compared using Student’s *t*-test. Nonparametric continuous variables were expressed as median (interquartile range (IQR)) and compared using the Mann–Whitney *U* test, and categorical variables were expressed in percentages and compared using the Pearson’s χ^2^ test. Spearman’s correlation was used to perform a trend test between tertiles of colloids and AKI.

Because colloids are indicated for restoration of the circulating volume, they are given in situations associated with AKI in which it may be difficult to distinguish the effect of the colloid from the underlying condition. Adjustment for an indication bias was further assessed using a propensity score. This methodology permits the comparison of patients who received a colloid to controls with a similar AKI risk profile. We followed the Strengthening the Reporting of Observational Studies in Epidemiology guidelines [18]. The propensity score was obtained using every variable statistically associated with the prescription of the colloid of interest. These were used in a logistic regression in which each variable was forced into the final model with the suggested ratio of approximately 1:10 variable/event. We then derived, for each patient, the probability (propensity score) of receiving colloids and compared exposed to unexposed patients in a 1:1 ratio, matched by the closest propensity score up to a ±0.05 difference [19]. This suggested that caliper size was very similar to another proposed caliper size defined by a 0.2 standard deviation of the propensity score (see Results). Each control patient was matched to only one treated patient (that is, there was no replacement) [20]. We then assessed whether treated and untreated groups balanced in terms of predictors of AKI. We also repeated this methodology in the subset of patients without hemodynamic instability as defined by a postoperative cardiovascular SOFA score of zero.

In the statistical analyses, we excluded cases with missing covariates. All *P*-values were two-tailed, and values <0.05 were considered statistically significant. Analyses were carried out using SPSS software (version 19; SPSS, Chicago, IL, USA).

## Results

### Study cohort

The characteristics of the 984 individuals included in the study are detailed in Table 1. The cohort characteristics included a majority of Caucasian men, mean age of 66 ± 10 years, normal preoperative renal function and a high prevalence of cardiovascular risk factors. Seventy-seven percent of individuals had a normal LVEF (≥50%), and 6.3% required the use of an IABP perioperatively.

**Table 1:** **Preoperative, perioperative and postoperative patient characteristics (** ***N*** **= 984)** ^**a**^

**Preoperative characteristics**		**Perioperative and postoperative characteristics**	
Age (yr)	66 ± 10	Use of intra-aortic balloon (%)	6.3
Female (%)	25	Heart valve surgery (%)	23
Caucasian (%)	94	CABG (%)	93
Active smoking status (%)	27	Duration of ECC (hr) (IQR)	1.1 (0.9 to 1.5)
Initial GFR (ml/min/1.73 m^2^)	72 ± 19	Urgent surgery (%)	14
Comorbidities		Reintervention (%)	4
Hypertension (%)	72	Postoperative cardiovascular SOFA score ≤2, >2 (%)	51, 49
Diabetes (%)	31	Perioperative transfusions	
Hyperlipidemia (%)	69	Red blood cells (0, 1, 2, ≥3 U) (%)	45, 15, 14, 22
Peripheral vascular disease (%)	8	Platelets (0, ≤10, >10 U) (%)	80, 17, 3
Stroke (%)	7	Fresh frozen plasma (0, ≤5, >5 U) (%)	80, 16, 4
MI within the past 7 days (%)	13		
LVEF ≤35%, 36% to 49%, ≥50% (%)	9, 14, 77	AKI (%)	
Drugs used within 5 days prior to surgery		AKIN stage 1	12.0
Diuretics (%)	34	RIFLE risk	5.3
RASB (%)	59	RIFLE injury	1.9
NSAIDs (%)	9	RIFLE failure	0.7
Contrast agent for angiography (%)	26		
Preoperative hemoglobin (g/L)	130 ± 17		

^a^Results are expressed as mean ± standard deviation, median (interquartile range (IQR)) or percentage as appropriate. AKI, acute kidney injury; AKIN, Acute Kidney Injury Network; CABG, Coronary artery bypass graft; ECC, Extracorporeal circulation; GFR, Glomerular filtration rate; LVEF, Left ventricular ejection fraction; MI, Myocardial infarction; NSAIDs, Nonsteroidal anti-inflammatory drugs; RASB, Renin angiotensin blockade; RIFLE, Risk, injury, failure, loss and end-stage kidney disease; SOFA, Sequential Organ Failure Assessment.

**Figure 1:**
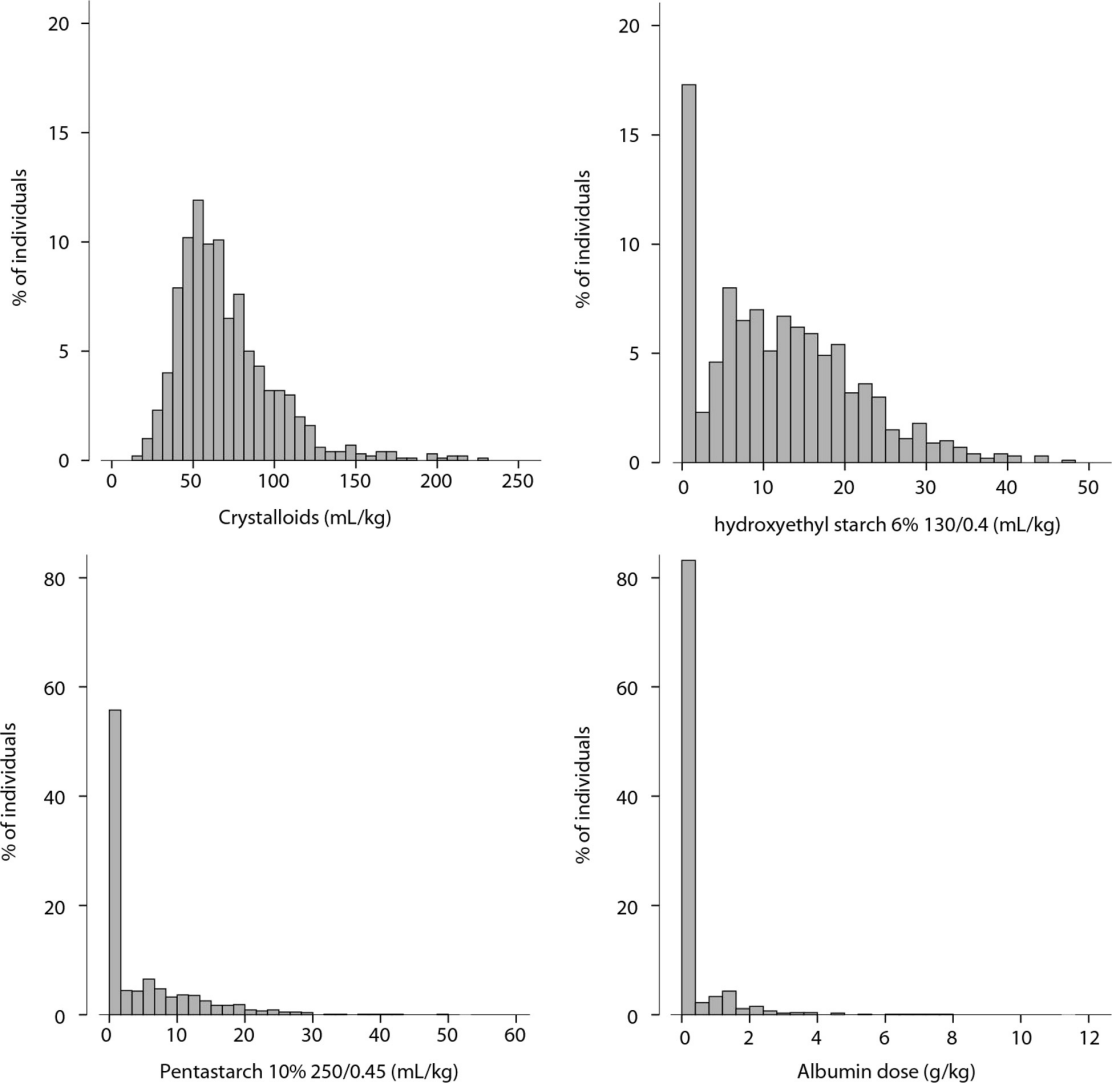
**Fluid administration from surgery until 36 hours postoperatively (** ***N*** **= 984).**

Fourteen percent of surgeries were defined as urgent, and twenty-three percent included valve replacement or repair. The median duration of ECC was 1.1 hour (IQR, 0.9 to 1.5). The median volume of crystalloids received from the beginning of surgery until 36 hours postoperatively was 63 ml/kg (IQR, 49 to 83). Eighty-two percent, forty-three percent and sixteen percent of individuals received HES 6%, pentastarch 10% or albumin, respectively, with median volumes of 13 ml/kg (IQR, 8 to 19) and 9 ml/kg (IQR, 6 to 15) and a median dose of 1.4 g/kg (IQR, 1.1 to 2.2), respectively (Figure 1). RBCs were given to half of the patients, and FFP and platelets were both given to 20% of patients. Half of the patients had a cardiovascular SOFA score >2 after surgery.

**Figure 2:**
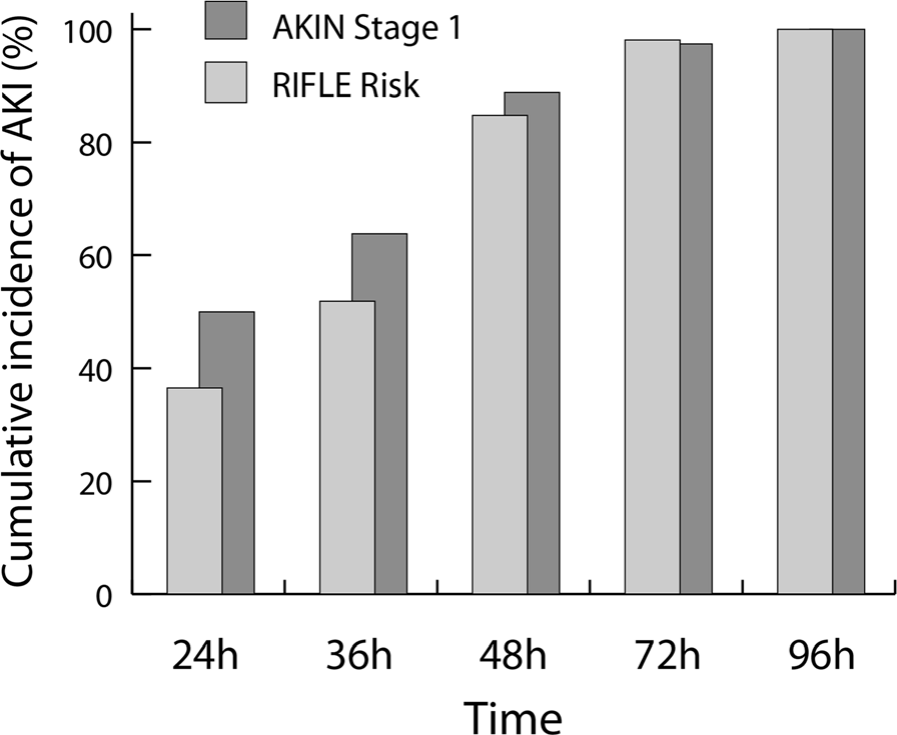
**Cumulative risk of acute kidney injury during the 96-hour postoperative observation period.** AKI, Acute kidney injury; AKIN, Acute Kidney Injury Network; RIFLE, Risk Injury Failure Loss and End-stage kidney disease.

Within 96 hours following surgery, RIFLE risk for AKI occurred in 5.3% of patients, RIFLE injury occurred in 1.9% and RIFLE failure occurred in 0.7%, whereas the incidence of AKIN stage 1 was 12.0%. No patient received RRT within 96 hours following surgery. By 36 hours after surgery, 52% of AKI defined by RIFLE risk and 64% of those defined by AKIN stage 1 had occurred (Figure 2).

### Predictors of acute kidney injury following cardiac surgery

**Figure 3:**
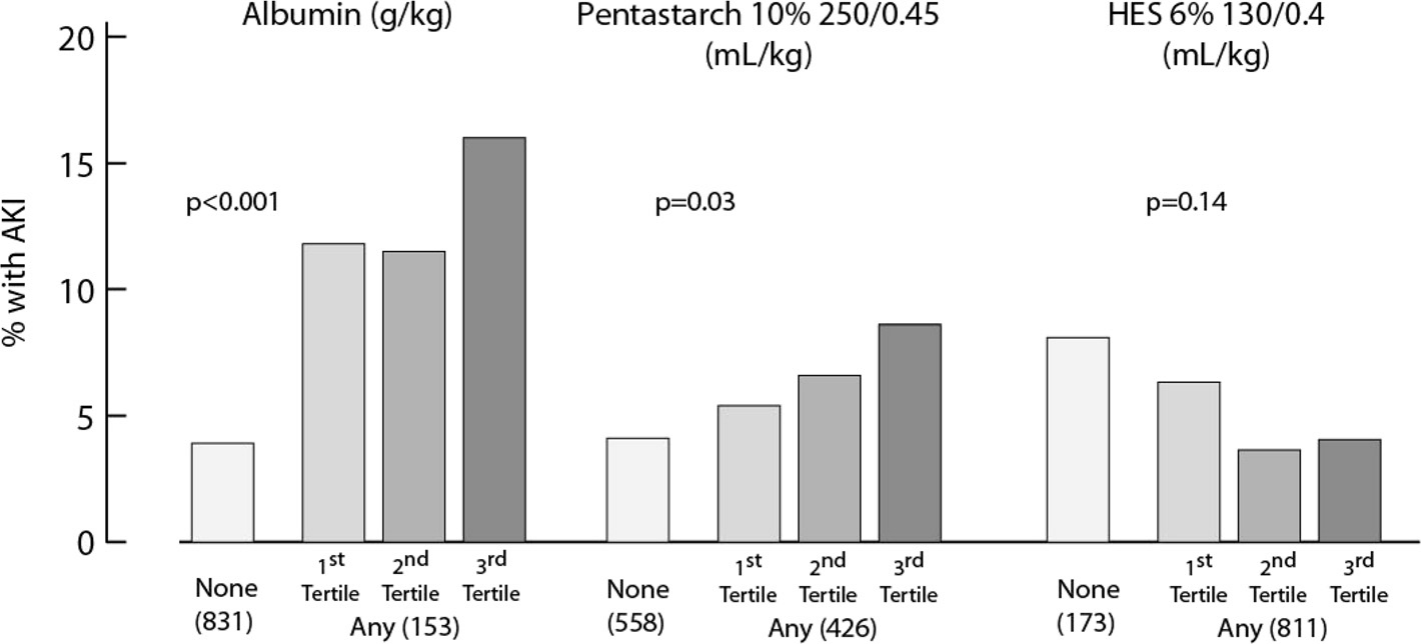
**Univariate dose–response risk for acute kidney injury using colloids.** The number of patients per category is shown in parentheses. Spearman’s correlation was used to test the association between tertiles of colloids and acute kidney injury (AKI). HES, Hydroxyethyl starch.

Risk factors for AKI using the RIFLE serum creatinine criterion are shown in Table 2. A preoperative reduced LVEF, lower hemoglobin and use of diuretics were predictive of AKI. Perioperatively, valvular surgeries, need for IABP, duration of ECC, cardiovascular SOFA score and number of RBCs, FFP transfusions and platelet transfusions were also associated with AKI. With regard to colloids, the greatest risk for AKI was associated with the use of albumin (odds ratio (OR), 3.9; 95% confidence interval (CI), 2.1 to 6.8; *P* < 0.001), marginal with the use of pentastarch 10% (OR, 1.7; 95% CI, 1.0 to 3.0; *P* = 0.06) and absent with the use of HES 6%. There was a dose-dependent risk for AKI with the use of albumin and pentastarch 10% (Figure 3). This association was not seen with HES 6% or crystalloids. Sex, age, ethnicity, smoking status, prior history, initial GFR, other medications, urgent surgery and need for reintervention were not statistically associated with AKI.

**Table 2:** **Variables associated with acute kidney injury as defined by RIFLE risk** ^**a**^

	**No AKI (** ***n*** **= 932)**	**AKI (** ***n*** **= 52)**	***P*** **-value**
Preoperative LVEF, ≤35%, 36% to 49%, ≥50% (%)	9, 13, 78	15, 21, 64	0.02
Preoperative use of diuretics (%)	33	56	0.001
Preoperative hemoglobin (g/L)	130 ± 17	123 ± 22	0.002
Heart valve surgery (%)	22	37	0.01
Duration of ECC (hr)	1.21 ± 0.62	1.59 ± 0.79	<0.001
Use of intra-aortic balloon pump (%)	6	14	0.03
Postoperative cardiovascular SOFA score 0 or 1, 2, 3 or 4 (%)	51, 0, 49	27, 2, 71	<0.001
Any perioperative transfusions (%)			
Red blood cells	50	79	<0.001
Fresh frozen plasma	20	35	0.005
Platelets	19	37	<0.001
Any pentastarch 10% received^b^ (%)	43	56	0.06
Any albumin received (%)	14	38	<0.001

^a^Results are expressed as mean ± standard deviation, median (interquartile range) or percent, as appropriate. ^b^The dose of pentastarch was associated with acute kidney injury (AKI) (see Figure 3). Sex, age, ethnicity, smoking status, prior history, initial estimated glomerular filtration rate, other medication, urgent surgery, need for reintervention and amount of crystalloids and hydroxyethyl starch 6% were not statistically associated with AKI. ECC, extracorporeal circulation; LVEF, left ventricular ejection fraction; SOFA, Sequential Organ Failure Assessment score.

Using the AKIN stage 1 definition of AKI, we identified the same predictors we did with the RIFLE risk. In addition, older age, lower initial GFR, history of diabetes, history of hypertension and preoperative renin angiotensin blockade were associated with AKIN stage 1 (data not shown).

### Risk of acute kidney injury with albumin administration in propensity-matched patients

The adjustment for selection bias was further addressed using a propensity score. Because albumin administration was identified as a novel risk factor of AKI following cardiac surgery, we further studied this finding by pairing those who received albumin with similar individuals who did not. Albumin administration was associated with multiple clinical variables, including age, LVEF, heart valve surgery, GFR, cardiovascular SOFA score, fluids (crystalloids, HES 6% and pentastarch 10%), transfusions (RBCs, FFP and platelets), preoperative hemoglobin, duration of ECC and use of diuretics. We derived the probability of receiving albumin (propensity score) with logistic regression using variables associated with its use, and then we matched individuals with a similar risk profile (propensity score ±0.05) who either received albumin or did not. Our ratio of variable/event was 8.3. Using a caliper size of 0.2 times, the standard deviation of the propensity score would have been similar at 0.042. Within the group of 153 individuals given albumin, we could match 141 of them to 141 similar controls. Risk factors for AKI between these groups were balanced, including the amount of fluid administered (82 ± 35 ml/kg without albumin compared to 78 ± 30 ml/kg with albumin, *P* = 0.28). The only exception was the volume of pentastarch 10%, which was lower in the albumin group (Table 3). Despite this, the risk for AKI associated with the use of albumin remained statistically higher (RIFLE risk: 12% versus 5%, *P* = 0.03; AKIN stage 1: 28% versus 13%, *P* = 0.002).

**Table 3:** **Risk of acute kidney injury with albumin administration in propensity-matched patients** ^**a**^

	**No albumin (** ***n*** **= 141)**	**Any albumin (** ***n*** **= 141)**	***P*** **-value**
Age (yr)	70 ± 9	69 ± 9	0.81
GFR (ml/min/1.73 m^2^)	62 ± 18	62 ± 20	0.78
LVEF ≤35%, 36% to 49%, ≥50% (%)	14, 11, 75	13, 19, 68	0.13
Preoperative hemoglobin (g/L)	127 ± 18	127 ± 19	0.99
Diuretics (%)	50	50	1.00
Heart valve surgery (%)	35	31	0.45
Duration of ECC (hr)	1.5 ± 0.7	1.6 ± 0.8	0.94
Cardiovascular SOFA score (immediately postoperatively) ≤2, >2 (%)	34, 66	38, 62	0.52
Crystalloids perioperatively until 36 hr postoperatively^b^ (ml/kg)	82 ± 35	78 ± 30	0.28
Any HES 6% perioperatively until 36 hr postoperatively (%)	85	91	0.10
Dose of HES 6% received^b^ (ml/kg)	16 (9 to 23)	14 (8 to 20)	0.30
Any pentastarch 10% perioperatively until 36 hr after surgery (%)	38	36	0.71
Dose of pentastarch 10% received^b^ (ml/kg)	13 (8 to 18)	8 (5 to 16)	0.03
Red blood cell transfusions, 0, 1, 2, 3+ U (%)	30, 9, 15, 45	19, 16, 20, 45	0.07
Fresh frozen plasma transfusions, 0, ≤5, >5 U (%)	67, 28, 5	67, 23, 10	0.70
Platelet transfusions, 0, ≤10, >10 U (%)	73, 23, 4	71, 22, 7	0.59
AKI			
AKIN stage 1 (%)	13	28	0.002
RIFLE risk (%)	5	12	0.03
RIFLE injury (%)	1	5	0.09

^a^AKI, Acute kidney injury; AKIN, Acute Kidney Injury Network; ECC, Extracorporeal circulation; GFR, Glomerular filtration rate; HES, Hydroxyethyl starch; LVEF, Left ventricular ejection fraction; RIFLE, Risk, injury, failure, loss and end-stage kidney disease; SOFA, Sequential Organ Failure Assessment. ^b^The median (interquartile range (IQR)) value given applies to the subgroup that received the colloid.

With regard to the timing of AKI, both groups satisfied the creatinine criteria at the same time. In the propensity-matched group that received albumin, 20 stage 1 AKI events occurred before 36 hours, 15 occurred between 36 and 48 hours and 5 occurred after 48 hours. The group that did not receive albumin had 10 events before 36 hours, 9 between 36 and 48 hours and none thereafter. The proportions were similar in both groups. Had AKI diagnosed prior to 36 hours influenced the prescription of albumin, we would have expected a trend toward an earlier diagnosis of AKI in the albumin group.

### Risk of AKI with albumin administration in propensity-matched patients with a perioperative cardiovascular SOFA score of zero

We also addressed the subgroup of individuals without hemodynamic instability as defined by a perioperative cardiovascular SOFA score of zero (*n* = 490). Within this subgroup, we derived a new propensity score predictive of the use of albumin with the same defining variables that we used for the entire cohort. We were able to match 50 cases to 50 controls with a similar risk profile, all of whom had a perioperative cardiovascular SOFA score of zero (Table 4). There was a 57% patient overlap with the group described above. The risk factors for AKI also balanced out between the two groups, except for a lower dose of pentastarch and a higher rate of RBC transfusions in the albumin group. Even in this different population, there was a significantly higher risk of AKIN stage 1 AKI (30% versus 8%, *P* = 0.005).

**Table 4:** **Risk of acute kidney injury with albumin administration in propensity-matched patients with a perioperative cardiovascular Sequential Organ Failure Assessment score of zero** ^**a**^

	**No albumin (** ***n*** **= 50)**	**Any albumin (** ***n*** **= 50)**	***P*** **-value**
Age (yr)	70 ± 9	69 ± 9	0.81
GFR (ml/min/1.73 m^2^)	62 ± 19	63 ± 17	0.99
LVEF ≤35%, 36% to 49%, ≥50% (%)	10, 12, 78	5, 16, 79	0.78
Preoperative hemoglobin (g/L)	130 ± 16	127 ± 19	0.26
Diuretics (%)	46	46	1.00
Heart valve surgery (%)	24	18	0.47
Duration of ECC (hr)	1.2 ± 0.5	1.2 ± 0.5	0.63
Crystalloids perioperatively until 36 hr postoperatively^b^ (ml/kg)	76 ± 31	75 ± 26	0.89
Any HES 6% received perioperatively until 36 hr postoperatively (%)	84	90	0.38
Dose of HES 6% received^b^ (ml/kg)	16 (11 to 22)	12 (7 to 18)	0.10
Any pentastarch 10% perioperatively until 36 hr postoperatively (%)	50	48	0.84
Dose of pentastarch 10% received^b^ (ml/kg)	14 (9 to 20)	6 (4 to 15)	0.02
Red blood cell transfusions, 0, 1, 2, ≥3 U (%)	36, 14, 14, 35	11, 11, 37, 42	0.05
Fresh frozen plasma transfusions, 0, ≤5, >5 U (%)	69, 25, 6	58, 42, 0	0.21
Platelet transfusions, 0, ≤10, >10 U (%)	78, 20, 2	68, 32, 0	0.44
AKI			
AKIN stage 1 (%)	8	30	0.005
RIFLE risk (%)	4	12	0.14
RIFLE injury (%)	4.0	4.0	1.00

^a^AKIN, Acute Kidney Injury Network; AKI, Acute kidney injury; ECC, Extracorporeal circulation; GFR, Glomerular filtration rate; HES, Hydroxyethyl starch; LVEF, Left ventricular ejection fraction; RIFLE, Risk, injury, failure, loss and end-stage kidney disease; SOFA, sequential organ failure assessment score. ^b^The median (IQR) given applies to the subgroup that received the colloid.

## Discussion

In our present study, we found that albumin administration was associated with a dose-dependent increased risk of AKI in patients undergoing cardiac surgery. To further assess the risk associated with albumin and adjust for potential indication biases, we paired individuals who received albumin to controls who received no albumin. On the basis of this propensity score, albumin administration was still associated with a twofold increased risk for AKI. We repeated this methodology in individuals without significant postoperative hemodynamic instability and found similar results.

To our knowledge, this is the first study to show a dose–response relationship between albumin administration and increased risk for AKI. Our study offers new insights into the association between albumin administration and kidney function. Current evidence regarding the beneficial or deleterious effect of albumin in this context is inconclusive, especially in surgical patients and those without hypoalbuminemia [4,8-10,12,21-23]. In recent international consensus statements and guidelines on AKI and fluid administration [7,11], experts have recommended the use of crystalloids ahead of albumin in patients at risk for or with AKI and advised against the use of hyperoncotic albumin solutions for fluid resuscitation. Most of the evidence in support of these recommendations originates from the CRYCO Study Group [10]. In their observational study of 1,013 patients with shock, hyperoncotic albumin was associated with a fivefold increased risk of renal event, defined as a twofold increase in creatinine or need for dialysis [10]. The authors did not report any dose–response relationship between albumin administration and risk for AKI. Of note, patients with cirrhosis, the group from which most of the evidence regarding the beneficial effect of albumin on kidney function derives, were excluded from the study [8,22,23]. The results of other small studies have suggested a deleterious effect of albumin on kidney function, but they had limited statistical power [21,24-26] or involved very large doses of albumin (more than 1,100 g per patient) [24].

In contrast to these findings, other studies have shown a protective or neutral effect on kidney function due to albumin administration [4,9,12,27]. In a recent meta-analysis, the protective effect of albumin on kidney function seemed to be present in patients with cirrhosis [9]. In the largest RCT conducted to date on albumin administration in critically ill patients (the Saline versus Albumin Fluid Evaluation (SAFE) study), albumin 4% was proven to be safe in terms of mortality and severe AKI compared to crystalloids [4]. Severe AKI was defined as a creatinine level >300 μmol/L and/or need for dialysis. However, the safety profile of albumin was not consistent between subgroups, with patients with traumatic brain injury having increased mortality [28] and patients with sepsis having decreased mortality [29]. There were no reports on kidney function in the traumatic brain injury *post hoc* study [28] and no increased rate of severe AKI associated with albumin in the septic subgroup [29]. In comparison, the ALBIOS study investigators looked at the effect of hyperoncotic albumin in patients with severe sepsis and septic shock and did not find any difference in either mortality as the primary outcome or in severe AKI, defined as a creatinine level >300 μmol/L [12]. The authors also looked at the incidence of AKI based on RIFLE criteria in a *post hoc* analysis and did not find a difference between the two groups [12]. The timing of AKI and albumin administration were not defined in the study. Importantly, the ALBIOS study differed from the other studies, as albumin 20% was administered on a daily basis if the patient’s albumin level was below 30 g/L and not according to the clinical context [12]. In addition, the cumulative fluid balance was lower in the albumin group, which could have reduced mortality and morbidity in that group [30-32]. In our present study, we included in our propensity score analysis the amount of fluid received by 36 hours after surgery to address this bias.

As highlighted in previous studies, the effect of albumin administration seems to differ between subgroups [27-29]. In our present study, we included patients undergoing cardiac surgery, a population with very limited data and a different pathophysiological model of AKI than patients with severe sepsis [21,33,34]. In a recent systematic review, authors highlighted the need for additional studies on albumin administration in surgical patients [35]. In cardiac surgery, a small study suggested that albumin may have a deleterious effect on kidney function [21], whereas another found no difference [34]. In the most recent study on this subject, researchers compared pentastarch, 25% albumin (75 g) and Ringer’s lactate solution and did not find any differences in creatinine levels between the three groups [33]. Of note, cardiac surgery patients were excluded from the SAFE trial [4], and only 6% to 7% of patients in the ALBIOS study had elective surgery [12]. The type of surgery was not mentioned in the ALBIOS study.

Our study was not designed to investigate underlying biological mechanisms to explain the effect of albumin on kidney function. Postmortem examinations of a limited number of patients who received large doses of 25% albumin showed no evidence of abnormal albumin storage [36]. The results of basic science studies on the renal impact of albumin administration have been contradictory [37]. Albumin may have anti-inflammatory and antioxidant properties to protect against organ damage [35,38,39]. However, although there are a number of mechanisms by which albumin might exert a beneficial effect, they are as yet unproven. In opposition to popular belief, according to the Starling equation, glomerular filtration pressure decreases as intracapillary oncotic pressure increases more than hydrostatic pressure, a situation favored by the use of hyperoncotic colloids [37]. This principle may partly explain our findings regarding the dose-relationship effect between the risk of AKI and the amount of albumin administered. In light of recent findings supporting the beneficial effect of chloride-restrictive fluid administration on kidney function [40], we ensured that albumin solutions had similar or lower chloride concentrations compared to normal saline, and we confirm that the use of crystalloids with lower chloride content was minimal at our institution when the study was conducted.

Although our present study was focused on kidney function as a primary endpoint, perioperative kidney function is a very relevant parameter in predicting long-term outcomes [41-43]. AKI is an important independent predictor of postdischarge mortality in patients undergoing cardiac surgery [41-43]. Other large studies have confirmed these findings in a broader population [44,45] as well as the deleterious influence of AKI on the development of chronic kidney disease (CKD) and ESRD [45,46]. Even mild AKI, defined as an increase in serum creatinine by 27 μmol/L (0.3 mg/dl), is associated with an increase in mortality [42,44,47,48], development of CKD [49], increased length of stay [48] and costs [47]. Importantly, some of these studies were conducted in patients undergoing cardiothoracic surgery, as in our study population [42,48].

Our study has several strengths. It is the largest study to date on the effect of albumin administration on kidney function in patients undergoing cardiac surgery, a clinical setting with very limited data and a different pathophysiological model of AKI than severe sepsis or shock. We were able to demonstrate a twofold increase risk in AKI after adjustment with a propensity score that included baseline characteristics, surgical aspects, severity of illness scores and amounts of blood products, colloids and crystalloids administered. As recently shown, propensity scores may underestimate the true effect size compared to RCTs in critically ill patients [20]. The use of a propensity score in our study could therefore underestimate the effect of albumin on the risk of developing AKI. Importantly, we have shown, for the first time to our knowledge, a dose–response relationship between albumin administration and risk for AKI in this population.

Our study also has limitations inherent to its retrospective and single-center design. First, there is a concern regarding hemodynamic instability (and risk of AKI) and the need to optimize left ventricular end-diastolic filling pressure using colloids. However, 86% of surgeries were elective, and any unstable condition was likely to surface after ECC at the end of the surgery. Our propensity score included the postoperative cardiovascular SOFA score. In addition, we obtained similar results in patients who had cardiovascular SOFA scores of zero. Second, we assessed whether the use of albumin by clinicians wishing to avoid giving patients synthetic colloids in the setting of impending AKI could have been a consequence of early AKI rather than its cause. However, the timing of AKI was similar in both groups. Third, it is possible that the period between the onset of oliguria and the rise of creatinine was a window within which albumin may have been preferred over synthetic colloids, again in the setting of impending AKI. However, we would have expected at least a trend toward greater use of crystalloids in the albumin group; however, the doses of crystalloids were not statistically different between groups (and lower in absolute numbers in the albumin group), even when addressing diagnosis of AKI <36 hours, 36 to 48 hours and >48 hours after surgery separately. We also had limited data on albumin levels. Recent studies have suggested a relationship between hypoalbuminemia and risk for AKI [50,51]. In our center, the postoperative prescription of albumin does not rely on a preoperative serum albumin value, as albumin is not often measured. Whether this could still be a confounder remains unknown. Some authors have suggested that albumin administration may improve morbidity once the albumin levels are increased up to 30 g/L [39,52], whereas others did not report such results [12]. As the majority of our patients had nonurgent surgeries, it is unlikely that their baseline albumin levels would have been <30 g/L. We did not have data on emerging biomarkers for AKI. Current observational studies still rely on serum creatinine for making an AKI diagnosis, as these emerging biomarkers are not largely available even today. Furthermore, as mentioned, AKI, as defined by serum creatinine changes, is a relevant endpoint because it is strongly associated with increased mortality and morbidity. Finally, we cannot rule out that the association between albumin administration and AKI may have resulted from a selection bias. There also might be unknown confounding factors for which we were unable to adjust.

## Conclusions

We found that albumin administration was associated with a twofold increased risk for AKI after adjustment with a propensity score in cardiac surgery patients, a population with a different pathophysiological model of AKI than severe sepsis or shock and with limited data on the effect of albumin administration. The detrimental effect was proportional to the dose administered. The results of this study are in accordance with recent guidelines suggesting the use of crystalloid solutions for most patients with or at risk for AKI [7,11]. Future RCTs should address the safety of albumin in terms of dose and concentration compared to crystalloids in cardiac surgery patients.

## Key messages

- In a retrospective cohort study including 984 patients undergoing cardiac surgery, we found that albumin administration was a risk factor for AKI.
- This finding remained significant when we used a propensity score methodology, even in patients without postoperative hemodynamic instability.
- We also identified a dose–response relationship between albumin administration and the risk for AKI.
- Future RCTs should address the safety of albumin in terms of dose and concentration compared to crystalloids in cardiac surgery.

## Abbreviations

AKI: Acute kidney injury
AKIN: Acute Kidney Injury Network
CABG: Coronary artery bypass graft
CKD-EPI: Chronic Kidney Disease Epidemiology Collaboration equation
ECC: Extracorporeal circulation
ESRD: End-stage renal disease
FFP: Fresh frozen plasma
GFR: Glomerular filtration rate
HES: Hydroxyethyl starch
IABP: Intra-arterial balloon pump
KDIGO: Kidney Disease: Improving Global Outcomes
LVEF: Left ventricular ejection fraction
RBC: Red blood cell
RCT: Randomized controlled trial
RIFLE: Risk, injury, failure, loss and end-stage kidney disease
RRT: Renal replacement therapy
SAFE study: Saline versus Albumin Fluid Evaluation study
SOFA: Sequential Organ Failure Assessment
